# Progressing haemorrhagic stroke: categories, causes, mechanisms and managements

**DOI:** 10.1007/s00415-014-7291-1

**Published:** 2014-03-05

**Authors:** Shiyu Chen, Liuwang Zeng, Zhiping Hu

**Affiliations:** Department of Neurology, Xiangya Second Hospital, Central South University, 139 Renmin Road, Changsha, 410011 Hunan People’s Republic of China

**Keywords:** Intracerebral haemorrhage, Progressing haemorrrahagic stroke, Early neurologic deterioration, Haematoma expansion, Perihaematomal oedema, Chronic encapsulated intracerebral haematoma

## Abstract

Haemorrhagic stroke is a severe stroke subtype with high rates of morbidity and mortality. Although this condition has been recognised for a long time, the progressing haemorrhagic stroke has not received adequate attention, and it accounts for an even worse clinical outcome than the nonprogressing types of haemorrhagic stroke. In this review article, we categorised the progressing haemorrhagic stroke into acute progressing haemorrhagic stroke, subacute haemorrhagic stroke, and chronic progressing haemorrhagic stroke. Haematoma expansion, intraventricular haemorrhage, perihaematomal oedema, and inflammation, can all cause an acute progression of haemorrhagic stroke. Specific ‘second peak’ of perihaematomal oedema after intracerebral haemorrhage and ‘tension haematoma’ are the primary causes of subacute progression. For the chronic progressing haemorrhagic stroke, the occult vascular malformations, trauma, or radiologic brain surgeries can all cause a slowly expanding encapsulated haematoma. The mechanisms to each type of progressing haemorrhagic stroke is different, and the management of these three subtypes differs according to their causes and mechanisms. Conservative treatments are primarily considered in the acute progressing haemorrhagic stroke, whereas surgery is considered in the remaining two types.

## Introduction

Haemorrhagic stroke, which accounts for 10–20 % of all of the new strokes that occur every year [78], has a 1-month mortality rate of approximately 40 % [164]. Although it has drawn the attention of researchers because of the high rates of morbidity and mortality, the outcomes and prognosis of intracranial haemorrhage have not improved significantly during the last several decades [142, 164].

Progressing stroke, also known as progressive stroke, stroke-in-progression, stroke-in-evolution, and deteriorating stroke, has been a clinical concept for a long time [27, 54]. Progressing stroke happens often within 36–72 h, with marked deterioration in clinical manifestations measured by the Scandinavian Stroke Scale or the Canadian Stroke Scale [83, 117, 154]. This concept was traditionally limited to ischaemic stroke [116], and haemorrhagic stroke was often ruled out in the initial studies of progressing stroke [84]. However, evidence has shown that a progression also exists in the haemorrhagic stroke [27, 54, 113]. Several researchers have shown that primary haemorrhagic stroke is more often associated with progression than ischaemic stroke [18] and that early deterioration is associated with a poorer outcome [93].

Early neurological deterioration of intracerebral haemorrhage has been recognised in many patients with haemorrhagic stroke [30, 46, 93, 100, 113]. As with progressing ischemic stroke, a deterioration of clinical signs and symptoms often happens within 24–72 h with intracerebral haemorrhage and is associated with haematoma expansion [30], perihaematomal oedema [46], intraventricular haemorrhage [100], and inflammation [155]. The expansion of haematoma may account for most of the progression [100, 113, 150]. Additionally, in the second to third weeks after the onset of intracerebral haemorrhage, many patients may undergo a deterioration of symptoms after the initial alleviation by conservative management, which indicates a subacute progression [171, 182]. Furthermore, the progression of haemorrhage may appear to be a chronic form, in which the gradual formation of an encapsulated intracerebral haematoma may cause progressive neurologic deficits over weeks or months [64, 69, 133]. Therefore, the concept of progressing haemorrhagic stroke should be separated from the progressing ischaemic stroke, because the causes, pathogenesis, mechanisms, manifestations and management of the former all differ from the latter.

In this review article, we proposed the concept of progressing haemorrhagic stroke and summarised the three categories of progressing haemorrhagic stroke, which are as follows: acute progressing haemorrhagic stroke, subacute progressing haemorrhagic stroke and chronic encapsulated intracerebral haematoma. We explored several aspects of their causes, mechanisms and management.

## Acute progressing haemorrhagic stroke

### Clinical features

Many researchers have observed an early neurological deterioration in spontaneous intracerebral haemorrhage. Despite the difference in the diagnostic criteria of early neurological deterioration, the studies have all shown a significant deterioration that occurs in 22.9–37 % of the patients [46, 100, 113, 150, 155]. In several retrospective studies, an ICH score of >2, white blood cell count of >10,000 cells/mL^3^, an initial Glasgow Coma Scale score of <14, large haemorrhage volume with significant mass effect on initial CT scan, haematoma expansion and intraventricular haemorrhage were shown to be associated with early neurological deterioration [46, 150, 155]. In a prospective study of 46 patients, Mayer et al. [113] proved that larger initial haemorrhages and marked initial mass effect predicted early neurological deterioration. A prospective study of 266 patients by Leira et al. [100] showed that on admission to the hospital, body temperature of 37.5 °C, increased neutrophil count and serum fibrinogen levels of >523 mg/dL could all independently predict neurological deterioration, whereas at 48 h, early ICH growth, intraventricular bleeding and highest systolic blood pressure were associated with neurological deterioration. Patients who experienced early neurological deterioration demonstrated significantly increased morbidity and mortality rates than those who did not [46, 100, 113]. The studies that focused on the early neurological deterioration are listed in Table 1.

**Table 1:** Early neurological deterioration occurrence in multiple studies

References	Type of study	Cases	END occur in	Factors associated with END
Mayer et al. [113]	Prospective	46	15 (33 %)	Causes: Haematoma enlargement, perihaematomal oedema, infarction; Predictive factors: Larger initial haemorrhages, marked mass effect
Flemming et al. [46]	Retrospective	61	16 (26 %)	Predictive factors: Glasgow Coma Scale score <14, Imaging characteristics such as haemorrhage volume >60 mL; shift of the septum pellucidum; effacement of the contralateral ambient cistern; widening of the contralateral temporal horn; Causes: Haematoma enlargement, perihaematomal oedema
Leira et al. [100]	Prospective	266	61 (22.9 %)	Predictive factors: On admission: Body temperature of 37.5 °C, neutrophil count, serum fibrinogen levels of 523 mg/dL; At 48 h: early ICH growth, intraventricular bleeding, highest systolic blood pressure
Sorimachi et al. [150]	Retrospective	184	19 (10 %)	Causes: Haematoma enlargement; hydrocephalus; convulsion; pneumonia
Sun et al. [155]	Retrospective	83	31 (37 %)	Predictive factors: Midline shift on imaging; ICH score; white blood cell count >10,000/mL^3^

*END* early neurologic deterioration

### Causes

#### Haematoma expansion

Haematoma expansion refers to the expansion of the haemorrhagic volume within the first 3–72 h, mostly within 6 h [50, 87]. Detected by neuroimaging methods, this expansion occurs in 13–40 % in all of the reported patients [20, 51, 88]. The definition of haematoma expansion is not universally agreed upon, and the commonly used definition is an increase in the volume of intraparenchymal haemorrhage of >33 % between the baseline and the repeated CT, increase of volume by ≥12.5 cm^3^ or by ≥1.4 times [20, 50, 87]. Haematoma expansion not only accounts for a major part of the acute progression in the acute phase of intracerebral haemorrhage [46, 100, 113], but is also independently associated with a poor outcome [20, 30, 32, 51].

#### Intraventricular haemorrhage

Intraventricular extension of haemorrhage is another deteriorating factor of early ICH [100, 155]. Intraventricular extension may occur simultaneously with ICH or within 24–72 h after the onset of initial ICH, in 20–55 % of all ICH patients [106, 152]. Steiner et al. [152] and Bhattathiri et al. [16] all showed that ICH patients with intraventricular haemorrhage had a worse functional outcome compared to those without intraventricular hemorrhage. Adjusting for the ICH score and haematoma expansion, intraventricular haemorrhage is still associated with a higher mortality rate within the patients’ hospitalisation stay [106].

#### Perihaematomal oedema

Perihaematomal oedema volume increases significantly after onset within the first 24 h after spontaneous ICH [56]. The chronological CT images showed that perihaematomal oedema increased rapidly within 3 days after onset and reached its initial peak in the fourth or fifth day [79, 165]. The highly evident initial mass effect could also contribute to the initial haemorrhagic stroke progression [46, 113, 182].

#### Inflammation

Sun et al. [155] reported that a white blood cell above 10,000/mL^3^ on hospital admission or within the first 72 h of hospital admission was highly associated with deterioration. Leira et al. [100] also showed that a body temperature of above 37.5 °C and increased neutrophil count are predictors of early neurological deterioration. The inflammation response predicts a worse short-term and long-term outcome [2, 36].

### Mechanisms

#### Blood clotting dysfunction

Continued haemorrhage from the primary haemorrhagic vessel or secondary bleeding into the periphery of the clot from the stretching of the surrounding vessels may account for the initial haematoma expansion or intraventricular haemorrhage [20, 43]. The ceaseless bleeding or re-bleeding in ICH may result from coagulopathy in certain patients. Haematoma expansion is shown to be positively associated with liver disease [87] and the amount of alcohol consumption [50], and negatively associated with the level of fibrinogen [50]. Warfarin use was associated with both haematoma expansion and intraventricular haemorrhage [17, 45, 47]. Broderick et al. and Yildiz et al. found a correlation between antiplatelet therapy and haematoma expansion [19, 180]. The low serum LDL cholesterol level was also reported to be associated with a higher haematoma expansion rate; researchers think that this association is related to the function of LDL to maintain vascular integrity [144].

#### Hypertension

Kazui et al. [87] showed that an interaction of hyperglycaemia and hypertension on hospital admission was associated with haematoma expansion. Takeda et al. [159] showed that blood pressure of >160 mmHg measured at 1.5 h after admission was significantly associated with haematoma expansion. Steiner et al. [152] also showed that increased baseline blood pressure was associated with intraventricular haemorrhage growth. Sykora et al. [156] showed that decreased baroreflex sensitivity was significantly correlated with increased blood pressure fluctuation and was an independent predictor of relative oedema. The antihypertensive therapies, which have become routine therapeutic methods in ICH, have received fair results in reducing haematoma enlargement, which will be described subsequently [8, 90, 137].

#### Hyperglycaemia

Hyperglycaemia on admission is an important predisposing factor for haematoma expansion [19]. Querish et al. [138] analysed the blood glucose of the ICH patients measured repeatedly after hospital admission over 3 days, and the results showed that those with increasing blood glucose had increased haematoma expansion and perihaematomal oedema, compared to those with decreasing blood glucose measurements. A linear correlation of intraventricular haemorrhage and hospital admission hyperglycaemia has also been detected by Appelboom et al. [9]. In an experimental model, Liu et al. [104] showed that hyperglycaemia increased haematoma expansion through the effect of increased kallikrein, which inhibits platelet aggregation. Hyperglycaemia may result from a history of diabetes, or stress reaction of ICH [170]. Many studies have shown that hyperglycaemia at the time of hospital admission is associated with early mortality and poor outcome in ICH patients [14, 89, 98, 170].

#### Haemorrhagic location

For patients with lobar ICH, an early mortality was associated with the involvement of the inferior parietal lobule, posterior insula and posterolateral thalamus, whereas for patients with basal ganglia ICH, early mortality was associated with a large region extending from the cortex to the brainstem [97]. Intraventricular haemorrhage extension is correlated with the primary location of ICH [152]. A retrospective study by Hallevi et al. [66] showed that thalamic and caudate locations had the highest intraventricular haemorrhage frequency. Lee et al. [97] also detected a higher incidence of intraventricular haemorrhage in the thalamic and basal ganglia. Intraventricular haemorrhage patients including the third and fourth ventricle or ICH patients with insular involvement are reported to have lower baroreflex sensitivity than the patients without these involvements, suggesting that involvement of these sites could contribute to impairment of autonomic blood pressure regulation [157, 158]. Hypertensive responses can be exaggerated and additive because of the impaired baroreceptor sensitivity [135].

#### Vasogenic oedema

Early perihaematomal oedema could be vascular in origin [22, 23]. Several animal experiments have confirmed that early oedema formation occurs despite an intact blood–brain barrier [168]. The oedema occurrence and the volume in thrombolysis-related patients with ICH are all less frequently observed than those seen in patients with spontaneous ICH, indicating that an existence of a clot is necessary for the presence of hyperacute oedema [55]. Blood clot retraction could force the serum into the perihaematomal space to form vasogenic oedema [178]. Butcher et al. [23] investigated 21 patients with ICH using perfusion-weighted MRI and diffusion-weighted MRI within 10 h; they found that water diffusion in the perihaematomal region was significantly increased and was independently correlated with perihaematomal oedema volume, and they suggested that the hyperacute oedema was, for the most part, plasma-derived.

#### Cytotoxic factors and inflammation

**Fig. 1:**
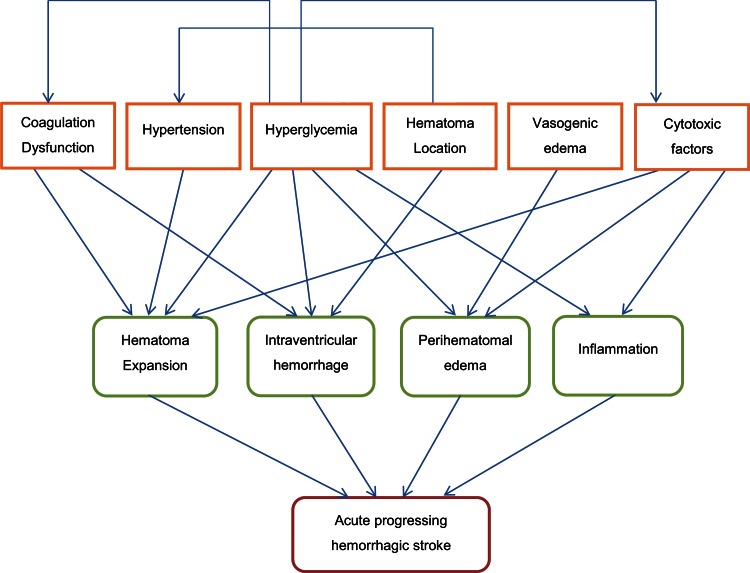
Mechanism of acute progressing haemorrhagic stroke

Thrombin, which is formed in the activated coagulation cascade in the early phase of ICH, primarily contributes to the development of early perihaematomal oedema [177] by activating Src kinase phosphorylation to destroy the blood–brain barrier via its protease-activated receptors [103]. Several animal studies have shown that thrombin could induce apoptosis of neurons and astrocytes [38], potentiate glutamate NMDA receptor function [10, 57], activate microganglia [119], activate autophagy process [71] or induce TNF-alpha release [73]. Also, the activated inflammation cascade may contribute to brain damage. Heme oxygenase, cellular fibronectin, interleukin-6, tumour necrosis factor-alpha, matrix metalloproteinase-9 (MMP-9) overexpression are all shown to be associated with haematoma expansion [48, 86, 149, 169]. Lee et al. [99] showed that by blocking the MMP-9 modulations in the experimental ICH of rats, a reduction of haematoma expansion can be observed (Fig. 1).

Each of the predisposing factors contributes to the pathogenesis of acute progressing haemorrhagic stroke, whereas several factors affect each other. The interaction of all causes promotes the progressing together.

### Prediction of acute progressing haemorrhagic stroke

The predisposing factors described all predict the progression of haemorrhagic stroke. Additionally, researchers have tried to develop more effective ways to predict the progressing haemorrhagic stroke. Because haematoma expansion accounts for the majority of acute progressing, the major explorations on predictions are on haematoma expansion. Several radiological methods have been developed.

Haematoma enlargement is less likely to occur in those who have a long interval (>6 h) from onset to first CT [12, 87]. ICH volume on baseline CT was positively associated with both haematoma expansion and intraventricular haemorrhage occurrence [66, 129]. A meta-analysis concluded that a smaller initial haematoma is less likely to expand [39].

Researchers have described the predictive radiological signs of the initial CT on admission to the hospital. A CT angiography ‘spot sign’ has proven to be an effective means for predicting haematoma growth [21, 35, 166]. The concept of the CTA ‘spot sign’ has evolved from the initial concept of contrast extravasation on postcontrast CT and was thought to represent ongoing bleeding [21, 101]. It was defined to be one or more foci of enhancement within the haematoma on CTA source images [166]. Four criteria have been proposed to identify the ‘spot sign’: (1) serpiginous or spot-like appearance within the margin of a parenchymal haematoma without connection to an outside vessel; (2) a contrast density of >1.5 mm in diameter in at least one dimension; (3) a contrast density (Hounsfield units, HU) of at least double that of the background haematoma; and (4) no hyperdensity at the corresponding location on non-contrast CT [163].

**Fig. 2:**
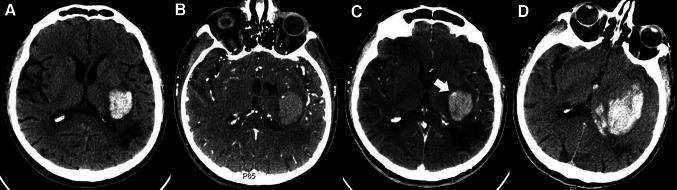
Patient with ‘spot sign,’ demonstrating extravasation and haematoma expansion [166]. Reproduced from Wolters Kluwer Health with permission

A multicentre, prospective, observational study has shown that the ‘spot sign’ significantly predicted haematoma expansion with a sensitivity of 51 % and specificity of 85 %, and was associated with a worse prognosis and increased mortality [35]. Several researchers have suggested that postcontrast CT extravasation could be an alternate to add the predictive value and sensitivity of spot sign [40, 65]. Furthermore, Almandoz et al. have developed a ‘spot sign score’ system, involving spot sign numbers, maximal axial dimension and maximal attenuation. The higher scores are associated with higher in-hospital mortality and poor outcome [33, 34]. A clinical trial is being conducted to test its predicting value in early haematoma growth [59] (Fig. 2).

**Fig. 3:**
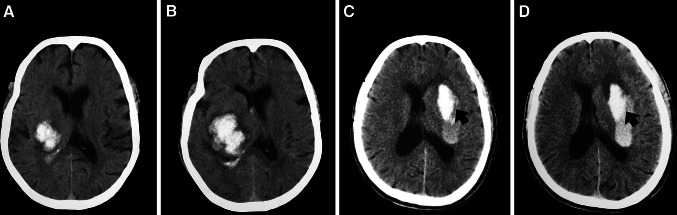
Haematoma heterogeneity and haematoma growth border. These two patients were admitted to our hospital with the impression of spontaneous ICH. Patient 1: **a** CT on admission shows haematoma heterogeneity with an initial haematoma volume of 15 mL. **b** Repeated CT after 32 h shows a haematoma volume of 30 mL. Patient 2: **c** CT on admission shows haematoma growth border (black arrow). **d** Repeated CT after 10 h shows haematoma expansion with increased midline shift

However, although ASA/AHA recommended CTA or contrast CT to identify the patients at risk for haematoma expansion [122], neither of them is a routinely performed examination at the time of hospital admission in many institutions. Haematoma density heterogeneity could be a substitute for the prediction of haematoma expansion. Haematoma heterogeneity refers to the irregularity of shape and density of the initial haematoma on the CT scan, and researchers have found an association between haematoma heterogeneity and haematoma expansion [13, 81]. Takeda et al. [159] concluded that the presence of haematoma volume above 16 mL, haematoma heterogeneity and 1.5 h of a systolic blood pressure above 160 mmHg together increased the likelihood of haematoma expansion to approximately 59 %. Although its definition was traditionally arbitrary, Ji et al. [81] defined the haematoma heterogeneity as a difference of over 20 HU in CT value between two regions exceeding 10 mm^2^ in area. Barras et al. used quantitative CT densitometry to measure mean attenuation, square root of variance, coefficient of variation, skewness and kurtosis of the attenuation distribution of the haematoma; they found that the coefficient of variation and the square root of variance, along with the basic haematoma volume, are predictors of greater growth. They suggested that quantitative CT densitometry can be used to identify haematoma heterogeneity [12]. Additionally, Ji et al. described a characteristic ‘haematoma enlargement border’, which was defined as an obvious boundary between high- and low-density regions within the primary haematoma on the CT. The haematoma enlargement border reflects on-going bleeding and its presence may be associated with potential haematoma expansion [81] (Fig. 3).

### Managements of acute progressing haemorrhagic stroke

#### Osmotherapy

Osmotherapy with mannitol is often applied in the acute phase of ICH to control the increased intracranial pressure. However, mannitol may have the possibility of aggregating haematoma expansion or perihaematomal oedema, because it may open the blood–brain barrier [29, 141] or reverse the osmotic concentration gradient between the oedematous brain and plasma [85]. In a randomised controlled trial, 128 patients with ICH were treated either with mannitol or sham infusion in their early stage of hospitalisation, and the result showed no difference in one-month mortality and secondary outcome [118]. The Cochrane systemic review, including two trials of mannitol for ICH, did not find significant differences in mortality and morbidity between treatment groups and controls [15]. In those without significant intracranial hypertension or mass effect, mannitol should not be used but close monitoring should be applied [136].

For a patient with an increased ICP, elevating the head to 30 degrees, giving 1.0–1.5 g/kg of 20 % mannitol by a rapid infusion, and hyperventilating the patient to a pCO_2_ of 28–32 mmHg are the usual management protocols [112]. Hypertonic saline is an alternative choice for decreasing intracranial pressure [91, 139]. Wagner et al. [167] treated ICH patients with spontaneous lobar and basal ganglia/thalamic bleeding with continuous hypertonic saline infusion within 72 h, and the relative oedema volume and the occurrence of intracranial pressure crisis had significantly decreased.

#### Blood pressure control

The protective effect of anti-hypertensive therapy in ICH has been broadly explored. A retrospective series of 76 patients by Ohwaki et al. showed that systolic blood pressure (SBP) of ≥160 mmHg was significantly associated with haematoma enlargement compared to those of ≤150 mmHg, and efforts to lower SBP below 150 mmHg may prevent this risk [130]. Also, several clinical trials have been conducted. The Intensive Blood Pressure Reduction in Acute Cerebral Hemorrhage Trial (INTERACT) randomised 404 patients to receive either standard blood pressure management (SBP <180 mmHg) or more aggressive blood pressure management (SBP <140 mmHg); the results showed a decrease in haematoma expansion by 2.80 mL, with no significant difference in perihaematomal oedema [8]. The results of the INTERACT phase 2 trail, performed on 2,839 patients, showed no significant reduction of haematoma expansion but significant improved functional outcome in the intensive blood pressure lowering group compared to the control group [7]. The Antihypertensive Treatment of Acute Cerebral Hemorrhage (ATACH) study randomised 60 patients to receive intravenous nicardipine hydrochloride to three systolic blood pressure reduction goals: 170–199, 140–169, or 110–139 mmHg. The post hoc analysis results showed that patients with a lower blood pressure were less likely to have haematoma expansion, perihaematomal oedema, and a poor 3-month outcome [137].

Presently, based on the existing incomplete evidence, AHA/ASA guidelines recommend that for patients with SBP of >200 mmHg or MAP of >150 mmHg, continuous intravenous infusion to reduce the BP should be applied, with BP monitoring every 5 min. For SBP of >180 mmHg or MAP of >130 mmHg in patients with a likelihood of ICP elevation, reducing BP while simultaneously maintaining a cerebral perfusion pressure of ≥60 mmHg is recommended. If there is no evidence of elevated ICP, then a modest reduction of BP (e.g., MAP of 110 mmHg or a target BP of 160/90 mmHg) with a re-examination every 15 min is recommended [121].

#### Coagulopathy reversal

Because coagulopathy contributes to the early progression of haemorrhagic stroke, ultra-early haemostatic therapies have been introduced to stabilise the condition and reduce the progressing [109]. For those patients who have been taking oral anticoagulants, such as warfarin, it is necessary to withdraw the drug and initiate a rapid reversal therapy, because it contributes to both a higher incidence of haematoma expansion [45, 47] and a higher mortality rate [145]. However, because warfarin may only contribute to rather than cause a role in the pathogenesis of ICH [146], discontinuing warfarin therapy with the administration of vitamin K is not sufficient. PCC [5, 24], rFVIIa [5], or FFP [60] are often considered [3, 4, 107]. Schlunk et al. [148] compared rFVIIa and PCC use in experimental warfarin-associated ICH and showed that they both could reduce the haematoma enlargement, with no significant difference in the reduction. A retrospective study showed that PCC was effective in reducing the fatality of warfarin-associated ICH [76]. Huttner et al. [77] retrospectively reviewed 55 patients who received PCC alone or in combination with FFP or vitamin K (*n* = 31), with FFP alone or in combination with vitamin K (*n* = 18), or with vitamin K as a monotherapy (*n* = 6), and the results showed that the incidence and the extent of haematoma growth were lower in patients receiving PCC compared with FFP and vitamin K. However, in a prospective research, de Leciñana et al. sought to detect the effect of anticoagulation reversal treatment in vitamin K antagonist-associated ICH, and the results showed that of anticoagulation reversal treatments—PCC with or without vitamin K, vitamin K monotherapy, or FFP with or without vitamin K—none were related to reduced mortality or functional outcome [31].

For anticoagulants, such as dabigatran, rivaroxaban or lepirudin, the previous antithrombotic therapy could also be applied, although there is no consensus for the treatment protocol [3, 95]. Because the role of antiplatelets in ICH has not been established, there is no recommended therapy for reversal, whereas the application of desmopressin acetate was reported by several researchers [3].

Recombinant activated factor VII for ICH has been explored in many studies. The Factor VII for Acute Intracerebral Hemorrhage phase IIB and phase III trials both showed that with an increased dose of rFVIIa, the haematoma growth volume decreased [110, 111]. However, the phase III trial showed that rFVII neither reduced mortality nor improved functional outcome [110]. The Cochrane systemic review, including six clinical trials with 975 patients receiving haemostatic drugs (two with epsilon-aminocaproic acid and 973 with rFVIIa), found no evidence of a reduction of death or dependence by haemostatic drugs [6]. Tranexamic acid is another hemostatic agent, and two multicentered clinical trials on tranexamic acid for ICH are presently recruiting patients [1, 115].

#### Blood glucose control

Although both diabetes and hyperglycaemia in non-diabetic patients were shown to have a higher early mortality, there are no agreed criteria of blood glucose control in the acute phase of ICH [131]. A prospective study by Godoy et al. [58] showed that insulin application within the first 12 h after ICH onset attenuated the association of hyperglycaemia with mortality. Ho et al. [70] selected 12 patients who underwent surgeries for spontaneous ICH and randomised them to intermittent or intensive continuous insulin infusion to maintain the blood glucose level between 4.0 and 8.0 mmol/L; the results showed that the continuous insulin infusion group had a lower MAP and ICP postoperatively. A randomised, controlled trial aimed at detecting whether 24 h of intensive glucose control by glucose-potassium-insulin infusions could reduce mortality or improve functional outcome failed to show a positive result, although the trial mainly focused on patients with ischemic stroke; the data of patients with ICH were not individually reported [62].

Presently, the AHA/ASA guidelines recommended that blood glucose should be maintained within normal range for patients with acute ICH [122].

#### Anti-inflammation therapy

Randomised trials have failed to demonstrate the efficacy of corticosteroids and, therefore, corticosteroid therapy is not recommended to treat patients with ICH [132, 161]. There are no other reported clinical trials that target the inflammatory cascade, although in several animal examinations, protease-activated receptor antagonists, iron chelators, N-acetylheparin, heme degradation inhibitors and antioxidants have been used [61, 74, 82].

#### Surgeries

Whether surgeries are beneficial for the patients with early haemorrhagic stroke has been controversial for a long time [42]. A retrospective analysis of Morioka suggested that surgically treated patients in the early stages of ICH demonstrated a better outcome [123]. The Surgical Trial in Intracerebral Hemorrhage (STICH) trial randomised 1,033 patients to receive either early surgery or conservative treatment, and the results showed no benefit of early surgery in patients with ICH [114]. However, the STICH trial neither individually explored those who are specifically thought to be more suitable for surgeries nor the results of certain types of surgery [112]. A meta-analysis involving 10 trials concluded that surgery added to medical management reduced the mortality rate and dependence after ICH [134]. Another meta-analysis that enrolled eight studies revealed that certain patients might benefit from surgery: within 8 h of onset, a volume of the haematoma between 20 and 50 mL, Glasgow Coma Score between 9 and 12, patients’ age range between 50 and 69 years, and superficial haematomas with no intraventricular haemorrhage [63].

The surgical methods include craniotomy, decompressive craniectomy, stereotactic aspiration, endoscopic aspiration, and catheter aspiration aimed at removing the clot [42, 63]. Certain types of surgeries may decrease the early progression of ICH. Rabinstein et al. [140] reviewed 26 patients with rapidly worsening symptoms with a significant mass effect who received craniotomy, and 22 % of the patients gained functional independence. Decompressive craniectomy is often combined with haematoma evacuation in treating ICH [160]. Fung et al. reviewed 12 patients who received decompressive craniectomy only, with 15 matched controls who were treated conservatively; three patients (25 %) of the treatment group died versus eight of 15 (53 %) of the control group, which shows feasibility for decompressive craniectomy without evacuation in treating ICH [53]. Because the clot and thrombin were the primary predisposing factors of the perihaematomal oedema development, Mould et al. conducted the Minimally Invasive Surgery and rtPA in ICH evacuation (MISTI) trial to evaluate the effectiveness of the clot lysis method. Their trial comprised 81 patients with minimally invasive surgery combined with recombinant tissue plasminogen activator administration and 42 patients with standard therapy; the results showed that both the haematoma volume and perihaematomal oedema were lower in the surgical group [124]. A meta-analysis by Zhou et al. involving 12 high-quality randomised, controlled trials concluded that minimally invasive surgery, especially stereotactic aspiration, could significantly reduce the early mortality of patients with ICH, and those with a superficial haematoma between 25 and 40 mL might most likely benefit from such surgeries [183].

For intraventricular extension of ICH, the removal of the blood clot in animal experiments showed benefits by controlling ICP, improving the level of consciousness and preventing tissue inflammation [67]. Compared to conservative therapy, extraventricular drainage, especially with fibrinolytic agents such as rtPA, significantly improved the case fatality rate and outcome [129]. rtPA may accelerate the clot lysis [126], but it also has the risk of aggregating bleeding. Naff et al. [125] tested removing the intraventricular haemorrhage with a catheter-delivered rtPA in the Clot Lysis: Evaluating Accelerated Resolution of IVH (CLEAR-IVH) trial; the results showed that the mortality rate, ventriculitis occurrence and bleeding events were all significantly lower in the treatment group, without significant changes in the systemic haematologic status [68].

#### Stroke unit

The observational study of Diringer and Edwards [37] showed that ICH patients admitted to a neuro intensive care unit is associated with reduced mortality rate, compared to those admitted to general ICU. Organized stroke care, or stroke unit, is effective in reducing mortality, institutionalization, and dependence in treating stroke patients [153], and it is recommended as a primary care model for stroke in many guidelines [41, 80, 128]. A recent systemic review including eight trials also showed that for ICH patients, stroke unit care was associated with a significant reduction of death or dependency [94].

## Subacute progressing haemorrhagic stroke

### Clinical features

Several of the patients with intracerebral haemorrhage develop late-onset deterioration, which often occurs during the second and third weeks. Although complications such as re-bleeding, deep vein thrombosis, and pneumonitis may occur, the subacute progression of haemorrhagic stroke should primarily be attributed to the increased mass effect produced by secondary oedema [11, 124]. As previously described, perihaematomal oedema has two peaks: in the fourth or fifth days, or during the second and third week [26, 165, 182]. This special feature of ICH is consistent with the progression of perihaematomal oedema after the onset of ICH [26, 79]. Subacute natural progression has not been reported much, and it is hard to identify in the beginning.

### Causes

#### Delayed perihaematomal oedema

Inaji et al. [79] investigated the chronological changes of perihaematomal oedema in 14 patients with CT scan and found that its volume increased rapidly within 3 days after haemorrhage, subsequently increased slowly until day 14, and decreased thereafter. Consistently, Venkatasubramanian et al. [165] investigated 27 patients prospectively with MR imaging and found that oedema volume growth was fastest in the first 2 days, and continued until 12 ± 3 days. By 2 weeks, the haemorrhage volume decreased, whereas oedema and haemorrhage plus oedema volumes significantly increased [11, 52]. A retrospective study involving 490 patients revealed that after admission to the hospital, the perihaematomal oedema increased, whereas the haematoma volume decreased, and the mass effect almost doubled between 7 and 11 days, which could cause an increase in ICP and secondary clinical deterioration [151].

#### Tension haematoma

**Fig. 4:**
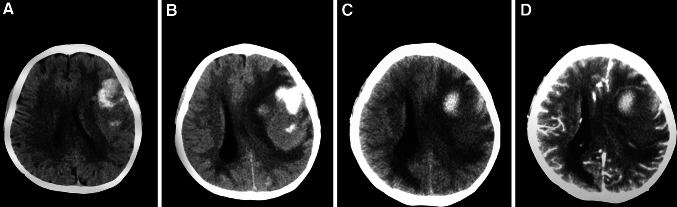
A case of subacute progressing stroke. The patient was admitted to our hospital because of dysphagia, hemiparalysis, and conscious disturbance. **a** CT on hospital admission showed left parietal and frontal intracerebral haemorrhage. After the initial treatment, the patient regained consciousness. **b** 10 days after hospital admission, the patient became lethargic, and repeated CT showed an increased mass effect and midline shift, with enlargement of the perihaematomal oedema. Stronger osmotic therapy was applied immediately and the patient’s consciousness improved. **c** Repeated CT at 17 days post hospital admission showed that the initial haematoma shrank further, the density of the brain oedema decreased, and the mass effect alleviated modestly. **d** An enhanced CT at 17 days showed no ring-enhancement, eliminating the tension haematoma

A specific ‘tension haematoma’, reported by Chinese scholars, might also develop in this phase. The patients often have a history of hypertension. The main features of tension haematoma are as follows: a sudden increase of ICP after the initial alleviation during conservative therapy, with a CT scan showing large regions of low density, isodensity or mixed-density near the initial haemorrhage [75]. A contrast CT could be used to distinguish perihaematomal oedema from tension haematoma. The ring-enhancement [172] on contrast CT at that time indicated the formation of tension haematoma, with regions interior to the ring-enhancement demonstrated tension haematoma, whereas the regions exterior to the ring-enhancement demonstrated perihaematomal oedema [105] (Fig. 4).

### Mechanisms

#### Red blood cell lysis and iron toxicity

Different from the initial oedema, the delayed perihaematomal oedema is mainly caused by erythrocyte lysis [72, 177]. It has been observed in ICH patients that during the second week, the perihaematomal oedema increases in size simultaneously with the lysis of the clot [173]. In several animal experiments, the intracerebral infusion of packed erythrocytes to the ICH rats caused oedema 3 days later [176], whereas the intracerebral infusion of lysed erythrocytes caused oedema within 24 h [175, 176], similar to the infusion of haemoglobin [174]. Furthermore, the upregulation of the primary heme degradation enzyme, heme oxygenase-1 (HO-1), has been observed in experimental ICH [169], simultaneously inhibiting HO-1 attenuated the brain oedema [174]. The infusion of haemoglobin degradation products, such as heme and Fe^2+^, could also induce brain oedema, whereas the oedema caused by the haemoglobin infusion could be attenuated by the infusion of iron chelator deferoxamine, indicating that iron degraded from haemoglobin is the primary cytotoxic factor that contributed to the delayed brain oedema [74].

#### Granulation tissue formation

Formation of tension haematoma is related to the granulation tissue around the initial haematoma. During the absorption of a haematoma, granulation tissues form around it, which can be seen on contrast CT as ring-enhancement [172]. The granulation tissue is capsule-like which limits the absorption of haematomas. Subsequently, the oncotic pressure inside the haematoma increases and the infiltration of plasma increases the tension inside the capsule progressively [162]. Additionally, blood may leak from the abundant capillaries contained in the granulation tissue repeatedly. Thus, this severely increased tension inside the initial haematoma cavity contributes to rapidly increasing ICP and deterioration of clinical conditions [105].

### Managements

#### Osmotherapy

When a sudden deterioration of clinical signs and symptoms occur during the conservative treatment process, a subacute progression of ICH should be suspected. A repeated CT at this time is necessary to determine whether there is increased mass effect or re-bleeding. The priority is to control the ICP, and a more intensive osmotherapy should be applied. Placement of an ICP monitor is recommended, especially in patients with a Glasgow Coma Scale score of less than eight and those with transtentorial herniation [122]. Several researchers have recommended an early and continuous infusion of hypertonic saline to reduce the impending perihaematomal oedema and progression of mass effect [167]. However, osmotherapy might not be beneficial for certain patients; therefore, surgery would be the best recommendation for these patients.

#### Surgeries

**Fig. 5:**
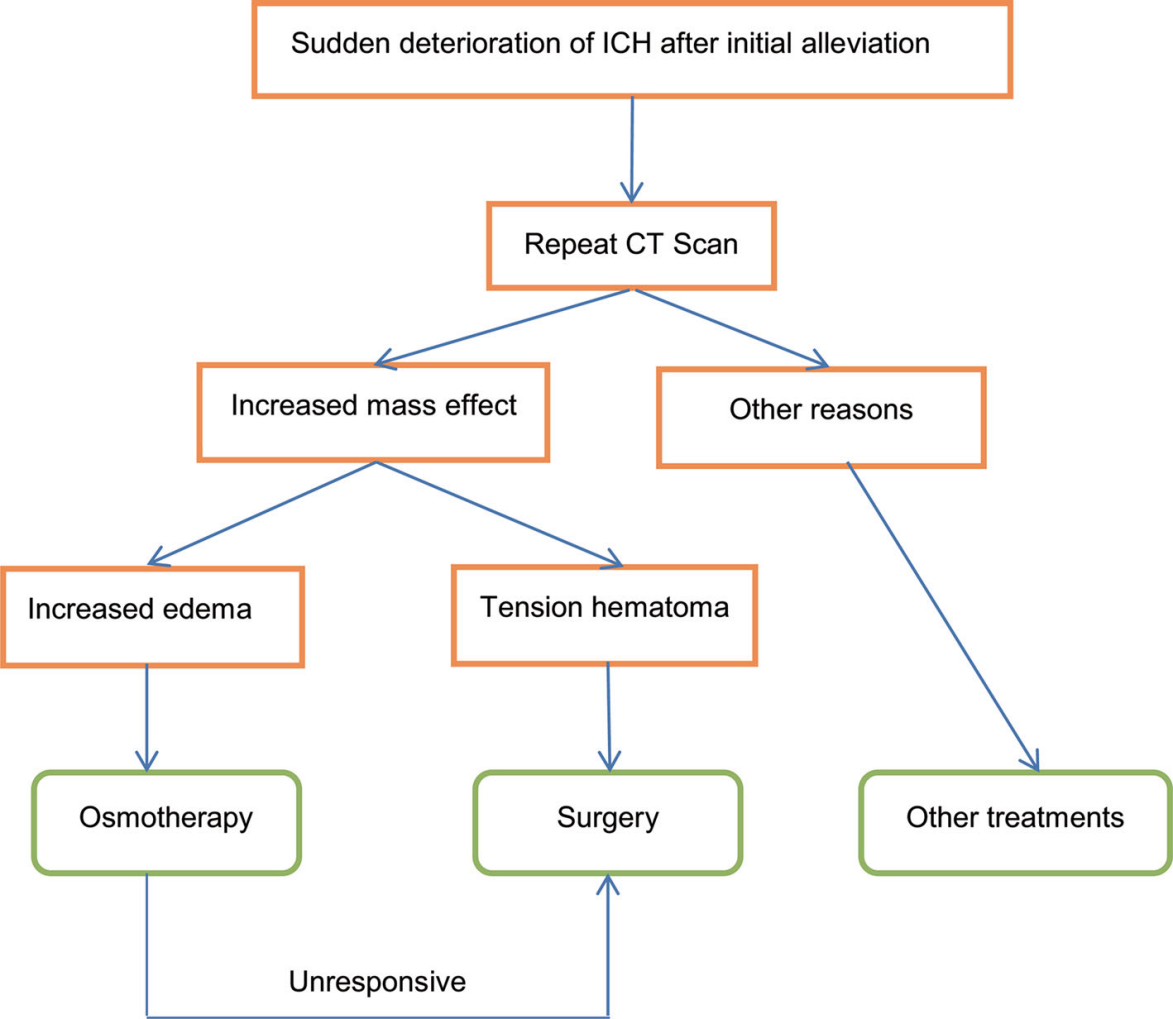
Management of subacute progressing stroke

As previously described, minimally invasive surgery is more applicable in reducing the perihaematomal oedema progression [124]. For the patients with tension haematoma, surgery is the only way to relieve the severely increased tension inside the haematoma [105]. If the hyperosmolar therapy was ineffective, a tension haematoma should be suspected. Once CT or MRI confirms the presence of a tension haematoma and markedly increased the mass effect, surgery is needed immediately to rescue the patient from rapidly increasing intracranial pressure. Such patients may benefit from stereotactic aspiration due to the highly increased tension inside the haematoma cavity [75, 105] (Fig. 5).

## Chronic progressing haemorrhagic stroke: chronic encapsulated intracerebral haematoma

### Clinical features

Chronic encapsulated intracerebral haematoma or chronic expanding intracerebral haematoma (CEICH) was first described by Hirsh in 1981 [69]; although it has been known for a long time, its causes and mechanisms have not been established. Different from spontaneous ICH, CEICH is characterised by a slow and gradual onset. The symptoms often evolve and appear within weeks or months, including seizures, progressive neurological deficit, such as mental disturbance, ataxia, hemianopia or hemiparesis, symptoms of increased intracranial pressure such as nausea, vomiting, headache, and papilloedema [108, 133, 147].

Neuroimaging strategies help to diagnose CEICH. The characteristic CT findings are round or similar round lesion with variable density, with or without calcification [28], and usually produce significant perilesional oedema and mass effect [25, 133, 147, 181]. Therefore, CEICH resembles an intracranial tumour or brain abscess and was initially easily misdiagnosed as those two [143, 181]. Characteristic MRI findings include: high-signal lesions indicating chronic haemorrhage with mixed-signal, which indicates recent haemorrhage on both T1 and T2 weighed images; low-signal surroundings on T2 images suggesting a fibrous encapsulation around the lesion [181]. Digital subtraction angiography is useful in detecting some of the underlying causes of CEICH [92, 127].

### Causes

For the encapsulated haematoma or CEICH, occult vascular malformations are often the underlying cause. Arteriovenous malformations [133], cavernomas [64, 120], microaneurysms, vascular amyloidosis or atherosclerosis are the common underlying causes [108, 133, 147]. CEICH also occurs after radiosurgeries [92] or trauma [25, 181]. Several causes remain unknown [147].

Whether hypertension contributes to the causes of CEICH is controversial. Most of the cases reported do not have a history of hypertension [64, 120], whereas several cases have been reported to be likely caused by hypertension [102]. Liquified chronic intracerebral haematomas is often secondary to hypertension [179] (Table 2).

**Table 2:** Causes of CEICH

Etiology	References
Hypertension	Yashon et al. [179] Lin et al. [102]
Arteriovenous malformation	Hirsh et al. [69] Pozzati et al. [133] Sakaida et al. [147]
Cavernoma	Masuzawa et al. [108] Monma et al. [120] Greiner-Perth et al. [64]
Radiosurgery	Kurita et al. [92] Lee et al. [96] Nakamizo et al. [127] Foroughi et al. [49]
Trauma	Yuguang et al. [181] Cakir et al. [25]
Unknown	Pozzati et al. [133] Fiumara et al. [44]

### Mechanisms

The mechanism of the progression of CEICH has not been established, and it is hard to predict whether CEICH will affect certain patients. On histological examination, CEICH consists of central haematoma and peripheral thick capsule. The haematoma often contains abundant blood clots at varied stages of development, hemosiderin-laden macrophages, cholesterol clefts, calcifications, fibrous tissue, and can present with or without arteriovenous malformations [102]. The capsule is composed of a thick outer membrane of dense collagen fibres and an inner thin granulation tissue rich in capillary [120, 127]. Researchers believed that some of the initial bleeding could stimulate a reactive response in the peripheral cerebral tissue and promote arachnoid fibroblast or collagen tissue to proliferate, forming the initial capsule membrane [44]; the subsequent repetitive bleeding or exudation produces granulation tissue and promotes the fibroblastic reaction to develop into a fibrous capsule [102, 181]. The blood elements leak into the cavity, causing the haematoma to expand [102].

In those patients without obvious vascular malformation in their histological examination, occult cerebrovascular malformations might account for the initial haemorrhage, which might be destroyed in the haemorrhagic episode [102, 143]. Pozzati et al. believes that the ‘self-destroying’ nature of the cerebral malformation primarily contributes to the formation of CEICH [133].

**Fig. 6:**
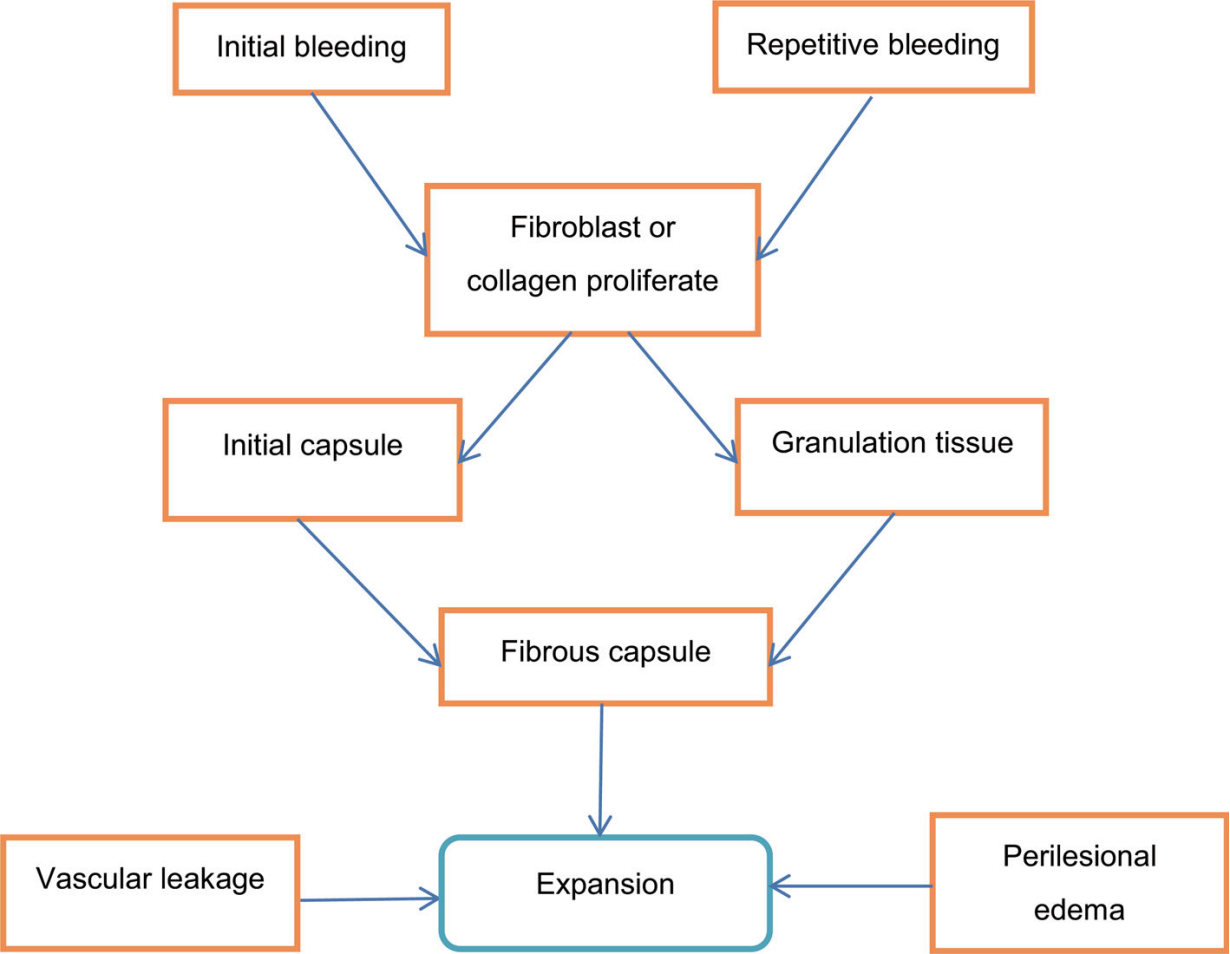
Mechanisms of CEICH

As for the radiosurgery, repetitive minor bleeding within the radionecrotic brain tissue most likely may initiate the formation of CEICH [49, 92, 96]. Nakamizo et al. [127] believes that radiosurgery could impose hypoxic stress on the surrounding brain tissue and induce a transcription of vascular endothelial growth factor, which could contribute to the abnormal angiogenesis and vascular leakage that expand the haematoma (Fig. 6).

### Managements of CEICH

Although spontaneous regression has been reported [64], most researchers recommend surgery for treating CEICH [127]. Because the recurrent bleeding of the capsule and abnormal vessels are the primary cause of the progression of CEICH, the capsule and hidden vessel malformation should also be evacuated as much as possible, as much as the haematoma itself, to avoid relapse and re-bleeding [181]. The entire lesion and the adjacent brain tissue should be carefully examined to rule out potential vascular malformation [64]. The prognosis of CEICH after surgery is usually good; however, patients could die from recurrent bleeding [133].

## Conclusions and future directions

In conclusion, we have proposed the concept of progressing haemorrhagic stroke, and we explored several aspects of its categories, causes, mechanisms and management. Despite tremendous efforts in this field, early deterioration and death are serious problems in patients with spontaneous intracerebral haemorrhage. Although some work has been conducted or is being performed to test the effectiveness of possible treatments, additional exploration of the mechanisms underlying the early deterioration is required. Furthermore, because of the special second oedema peak in ICH, subacute haemorrhagic stroke is specific in haemorrhagic stroke. However, subacute haemorrhagic stroke has rarely been reported or explored, although it is an important clinical entity that deserves concern. The causes and mechanisms are not very well understood, and the standard treatments need to be established. Chronic encapsulated intracerebral haemorrhage has gained increasing attention not only because of the more frequent recognition but also because of the prevalence of radiosurgery application in treating neurological diseases. More effective ways to predict and prevent the occurrence of CEICH are warranted.

## Search strategy and selection criteria

We searched PubMed, China National Knowledge Infrastructure (CNKI) and the Cochrane Library. We also searched the reference lists of retrieved articles, and we cross-referenced. Search terms included “progressing stroke”, “progressive stroke”, “stroke-in-evolution”, “h(a)emorrhagic stroke”, “cerebral h(a)emorrhage”, “intracerebral h(a)emorrhage”, “spontaneous intracerebral h(a)emorrhage”, “early neurological deterioration”, “secondary neurological deterioration”, “chronic encapsulated intracerebral h(a)emorrhage”, “h(a)ematoma expansion”, “perih(a)ematomal (o)edema”, “intraventricular h(a)emorrhage”, “tension h(a)ematoma”, “inflammation”, “treatment”, and “management”. The search included both human and animal studies. Papers published in both English and Chinese from 1971 to 2013 were reviewed. Articles were selected on the basis of relevance to the topics covered in the Review. Where issues are controversial, evidence on both sides of the issue is given.

## Abbreviations

CT: Computed tomography
ICH: Intracerebral haemorrhage
CTA: Computed tomography angiography
PET-CT: Positron emission tomography-computed tomography
rFVIIa: Recombinant factor VIIa
FFP: Fresh frozen plasma
CEICH: Chronic encapsulated intracerebral haematoma
